# Identification and characterization of *Staphylococcus devriesei* isolates from bovine intramammary infections in KwaZulu-Natal, South Africa

**DOI:** 10.1186/s12917-018-1655-1

**Published:** 2018-11-03

**Authors:** Tracy Schmidt, Marleen M. Kock, Marthie M. Ehlers

**Affiliations:** 1 Allerton Provincial Veterinary Laboratory, KwaZulu-Natal Department of Agriculture and Rural Development, Private Bag X2, Cascades, 3202 South Africa; 2 Department of Medical Microbiology, University of Pretoria, Pretoria, 0001 South Africa; 3 Tshwane Academic Division, National Health Laboratory Service, Pretoria, 0001 South Africa

**Keywords:** *Staphylococcus devriesei*, Bovine mastitis, CoNS, MALDI-TOF MS, Biolog

## Abstract

**Background:**

Coagulase-negative staphylococci (CoNS) are among the leading bacterial causes of bovine mastitis in many dairy-producing countries. Among the challenges associated with the specific diagnosis of CoNS infections is the biochemical heterogeneity of the species in the genus and the unavailability of accurate, cost-effective and up-to-date diagnostic tests. A previous study investigating the diversity of CoNS associated with cases of bovine mastitis in South Africa, resulted in six CoNS isolates which could not be identified despite the use of a combination of different molecular assays. The identification and characterisation of the isolates was pursued further in this study.

**Results:**

The six CoNS isolates in question were identified by sequencing multiple housekeeping genes (*dna*J, *hsp*60, *rpo*B, 16S rRNA) and characterized through the use of matrix-assisted laser/desorption ionization time of flight mass spectrometry (MALDI-TOF MS) and the Biolog GEN III Microplate™ bacterial identification system. Sequencing of housekeeping genes identified the isolates as *S. devriesei*. This *Staphylococcus* species was only described in 2010 and this is the first report documenting the isolation of *S. devriesei* from cases of bovine IMIs in South Africa. Analysis of mass spectra generated by the six isolates showed intra-species variation which was also observed when evaluating the metabolic profiles of the isolates using the Biolog GEN III system. Neither the MALDI-TOF MS nor the Biolog database are currently populated with data relating to *S. devriesei*, resulting in the isolates not being identified, in the case of MALDI-TOF MS analysis, or mis-identified as was observed with the Biolog GEN III system.

**Conclusions:**

The phenotyping data collected during this investigation provides useful information concerning *Staphylococcus devriesei* which could be used to populate user system databases thereby ensuring the accurate identification of isolates in future. The availability of improved diagnostics will in turn facilitate studies to elucidate the epidemiology, pathogenicity and true prevalence of this species in dairy herds.

## Background

Coagulase-negative staphylococci (CoNS) are a common cause of bovine intramammary infections (IMIs) or mastitis, a disease condition which causes significant financial losses to the dairy industry worldwide [1, 2]. The importance of CoNS as mastitis pathogens has been disputed as infections caused by this group of bacteria are usually mild and remain subclinical [3]. However, this perception is changing as CoNS have emerged as one of the leading bacterial causes of IMIs worldwide, including South Africa [4–6]. Research emanating primarily from European countries, has provided evidence to suggest that some CoNS, for example *S. chromogenes*, are more specifically adapted to the bovine udder and are capable of causing more serious infections than some of the other species [7]. The importance of differentiating and identifying CoNS to species-level has become increasingly important but is currently limited by the availability of appropriate, cost-effective diagnostic tests [8].

The genus *Staphylococcus* currently comprises of 49 validly described species and 22 subspecies [9]. At least 24 different CoNS have been reported from bovine mastitis cases [2]. In order to phenotypically differentiate CoNS, a large number of biochemical tests need to be carried out which is both time consuming and costly [10, 11]. Manual and automated bacterial identification systems are commercially available to facilitate the identification of *Staphylococcus* species, however, many of these systems have been found to produce inaccurate results when testing veterinary isolates [11–13]. The reason for this is attributed to the limited number of veterinary strains evaluated and incorporated into the reference databases [14]. Variability in the expression of phenotypic characteristics and the occurrence of phenotypic variants may also lead to the misidentification of *Staphylococcus* species or subspecies [15–17]. Advances in the molecular field have seen the development and application of DNA-based methods for the differentiation and identification of staphylococcal species [10, 14, 18]. Since these methods do not rely upon gene expression they are not subject to the same pitfalls as phenotyping methods [14]. In general, DNA-based methods have been shown to have a higher discriminatory power and show greater reproducibility and typeability than conventional phenotyping methods [14]. The inclusion of low-quality DNA sequence data in databases may however present challenges [15, 19].

Amplification and sequencing of variable regions in the 16S rDNA gene is one of the most commonly applied approaches used to differentiate bacterial species [17, 20]. Some reports have suggested that partial 16S rDNA sequencing of staphylococcal isolates may not always be discriminatory enough at the subspecies level [10, 16, 20]. Other housekeeping gene targets that have been evaluated for their suitability in discriminating between *Staphylococcus* species and subspecies include: chaperone dnaJ (*dna*J) [21]; elongation factor Tu (*tuf*) [15], the 60 kDa heat shock protein (*hsp*60) [22, 23], glyceraldehyde-3-phosphate (*gap*) [24], RNA polymerase B (*rpo*B) [16, 25] and superoxide dismutase (*sod*A) [26].

An alternative, protein-based approach to species and subspecies-level bacterial identification which is gaining traction is matrix-assisted laser desorption/ionization time of flight spectroscopy (MALDI-TOF MS) [11]. This method examines the pattern of proteins directly from intact bacterial cells, generating a unique mass-spectral fingerprint for isolates [27]. Comparison with a spectral database facilitates the identification of isolates based on their cellular protein ‘fingerprints’ [28]. The ability of MALDI-TOF MS to correctly identify CoNS has been evaluated in both medical and veterinary laboratories and the technology has been found to provide accurate and highly reproducible results [29]. Furthermore, MALDI-TOF MS analysis is quick, technically easy to perform and cost-effective compared with many molecular identification techniques [30].

Our research group recently conducted an extensive study to characterize CoNS implicated in cases of bovine IMIs recovered from dairy herds across the province of KwaZulu-Natal (KZN), South Africa [31]. A combination of techniques including multiplex-PCR (M-PCR), MALDI-TOF MS and sequencing of the *tuf* gene were used to identify the CoNS isolates to species-level. Despite the combinatorial approach used, five CoNS isolates could not be identified. The purpose of this investigation was to characterize the isolates further and investigate the taxonomic standing of the isolates within the *Staphylococcus* genus. A sixth isolate, which showed only a 97% similarity to *S. haemolyticus* when comparing the *tuf* gene sequence with sequences in GenBank, was included with the five isolates.

## Results

### Phenotypic characterization of isolates

A summary of the preliminary phenotyping and molecular results of the six CoNS isolates is presented in Table 1. After 48 h of incubation, a small zone of clear hemolysis was evident around colonies of all the test isolates. Some variation in colony appearance was observed between the isolates. Colonies of isolates AN22, AN25, BN7 and FN2 were grey-yellow in color, whilst colonies of AN3 and AN19 were yellow-orange in appearance.

**Table 1:** Preliminary characterization results for the coagulase-negative *Staphylococcus* isolates recovered from bovine intramammary infections

Isolate reference (farm of origin)	Phenotyping assays		Multiplex PCR 1				Species-level identification of CoNS isolates			Antimicrobial susceptibility testing											
	Coagulase production	Pastorex StaphPlus test	16S rRNA genus-specific target	*S. aureus nuc* gene	*mec*A gene	*luk* S/F gene	Bovine M-PCR^a^	MALDI-TOF MS (score)^b^	*tuf* gene sequencing	Penicillin (10 IU)	Ampicillin (10 μg)	Amoxicillin/Clavulanate (20 μg / 10 μg)	Cefoxitin (30 μg)	Cephalothin (30 μg)	Tetracycline (30 μg)	Trimethoprim/Sulfamethoxazole (1.25 μg / 23.75 μg)	Moxifloxacin (30 μg)	Clindamycin (2 μg)	Erythromycin (15 μg)	Gentamicin (10 μg)	Streptomycin (10 μg)
AN3 (commercial farm A)	(−)	(−)	(+)	(−)	(−)	(−)	(−)	No reliable identification	^c^	R	R	S	S	S	S	S	S	S	S	S	S
AN19 (commercial farm A)	(−)	(−)	(+)	(−)	(−)	(−)	(−)	No reliable identification	^c^	S	S	S	S	S	S	S	S	S	S	S	S
AN22 (commercial farm A)	(−)	(−)	(+)	(−)	(−)	(−)	(−)	No reliable identification	^c^	S	S	S	S	S	S	S	S	S	S	S	S
AN25 (commercial farm A)	(−)	(−)	(+)	(−)	(−)	(−)	(−)	No reliable identification	^c^	R	R	S	S	S	S	S	S	S	S	S	S
BN7 (commercial farm B)	(−)	(−)	(+)	(−)	(−)	(−)	(−)	*S. lugdunensis* (1.845 and 1.727)	^c^	R	R	S	S	S	S	S	S	S	S	S	S
FN2 (commercial farm F)	(−)	(−)	(+)	(−)	(−)	(−)	(−)	No reliable identification	^d^	S	S	S	S	S	S	S	S	S	S	S	S

^a^The bovine M-PCR assay comprised of primers specific for: *S. chromogenes*, *S. epidermidis*, *S. haemolyticus*, *S. simulans*, *S. warneri* and *S. xylosus*

^b^MALDI-TOF MS interpretation: 2.00 to 2.299 = secure genus identification, probably species identification; 1.70 to 1.99 = probable genus identification; < 1.7 = not a reliable identification

^c^Amplicon sequence showed 99–100% similarity with data in GenBank (EU571021)

^d^Amplicon showed 97% similarity to *S. haemolyticus*; (+) = positive; (−) = negative; S = sensitive; R = resistant

The phenotypic characterization of the test isolates using the Biolog GEN III MicroPlate™ identification system (Biolog, USA) was carried out in triplicate for each test isolate. The phenotyping results are summarized in Table 2. All isolates were uniform in their ability to utilize: L-alanine, acetoacetic acid, citric acid, dextrin, D-fructose, D-galactose, D-gluconic acid, α-D-glucose, glycerol, L-lactic acid, D-maltose, methyl pyruvate, N-Acetyl-D-neuraminic acid, pectin, sucrose, D-trehalose and D-turanose.

**Table 2:** Metabolic fingerprints for six *Staphylococcus devriesei* isolates obtained using the Biolog GENIII Microplate™ identification system

Assay		AN3	AN19	AN22	AN25	BN7	FN2
Carbon sources	Dextrin	+	+	+	+	+	+
	D-Maltose	+	+	+	+	+	+
	D-Trehalose	+	+	+	+	+	+
	D-Cellobiose	b	b	b	b	b	b
	Gentiobiose	+	+	b	b	b	+
	Sucrose	+	+	+	+	+	+
	D-Turanose	+	+	+	+	+	+
	Stachyose	b	b	b	b	(−)	b
	D-Raffinose	(−)	(−)	b	b	(−)	b
	α-D-Lactose	b	+	+	+	+	+
	D-Melibiose	b	b	b	b	b	b
	ß-Methyl-D-Glucoside	+	b	(−)	(−)	b	+
	D-Salicin	b	(−)	(−)	(−)	(−)	(−)
	N-Acetyl-D-Glucosamine	+	b	b	+	+	+
	N-Acetyl-β-D-Mannosamine	+	b	b	+	b	+
	N-Acetyl-D-Galactosamine	(−)	(−)	(−)	(−)	(−)	(−)
	N-Acetyl-Neuraminic Acid	+	+	+	+	+	+
	α-D-Glucose	+	+	+	+	+	+
	D-Mannose	b	b	b	b	b	+
	D-Fructose	+	+	+	+	+	+
	D-Galactose	+	+	+	+	+	+
	3-Methyl Glucose	b	b	b	b	b	b
	D-Fucose	b	b	b	b	b	b
	L-Fucose	b	b	b	b	b	b
	L-Rhamnose	b	b	b	b	b	b
	Inosine	b	+	+	+	+	+
	D-Sorbitol	b	b	b	b	b	b
	D-Mannitol	+	b	+	+	+	+
	D-Arabitol	+	+	b	b	+	+
	myo-Inositol	(−)	(−)	(−)	b	(−)	b
	Glycerol	+	+	+	+	+	+
	D-Glucose-6-PO4	b	b	b	b	b	b
	D-Fructose-6-PO4	b	b	b	b	b	b
	D-Aspartic acid	b	b	(−)	b	(−)	b
	D-Serine	(−)	(−)	(−)	(−)	(−)	b
	Gelatin	b	b	(−)	(−)	(−)	b
	Glycyl-L-Proline	(−)	+	b	(−)	b	b
	L-Alanine	+	+	+	+	+	+
	L-Arginine	+	+	(−)	(−)	(−)	+
	L-Aspartic Acid	b	b	(−)	b	(−)	+
	L-Glutamic Acid	+	+	b	b	+	+
	L-Histidine	(−)	(−)	(−)	(−)	(−)	(−)
	L-Pyroglutamic Acid	(−)	(−)	b	b	b	+
	L-Serine	+	+	(−)	+	+	+
	Pectin	+	+	+	+	+	+
	D-Galacturonic Acid	b	b	b	b	b	b
	L-Galactonic Acid Lactone	b	b	b	b	b	b
	D-Gluconic Acid	+	+	+	+	+	+
	D-Glucuronic Acid	b	b	b	b	b	b
	Glucuronamide	b	b	b	b	b	b
	Mucic Acid	(−)	(−)	(−)	b	b	b
	Quinic Acid	(−)	(−)	(−)	(−)	(−)	b
	D-Saccharic Acid	b	b	(−)	b	b	b
	*p*-Hydroxy-Phenylacetic Acid	(−)	(−)	(−)	(−)	(−)	(−)
	Methyl Pyruvate	+	+	+	+	+	+
	D-Lactic Acid Methyl Ester	b	b	b	b	b	b
	L-Lactic Acid	+	+	+	+	+	+
	Citric Acid	+	+	+	+	+	+
	α-Keto-Glutaric Acid	(−)	(−)	b	b	b	b
	D-Malic Acid	(−)	(−)	(−)	(−)	(−)	(−)
	L-Malic Acid	b	b	+	+	+	+
	Bromo-Succinic Acid	(−)	(−)	b	(−)	(−)	b
	Tween 40	b	+	b	b	b	+
	γ-Amino-Butyric Acid	(−)	(−)	(−)	(−)	(−)	(−)
	α-Hydroxy-Butyric Acid	b	+	+	b	+	+
	β-Hydroxy-D,L-Butyric Acid	(−)	(−)	(−)	(−)	(−)	(−)
	α-Keto-Butyric Acid	+	b	+	b	b	b
	Acetoacetic Acid	+	+	+	+	+	+
	Propionic Acid	+	b	b	+	b	+
	Acetic Acid	+	+	b	+	b	+
	Formic Acid	+	(−)	+	+	+	+
Chemical sensitivity assays	pH 6	+	+	+	+	+	+
	pH 5	b	b	(−)	(−)	(−)	b
	1% NaCl	+	+	+	+	+	+
	4% NaCl	+	+	+	+	+	+
	8% NaCl	+	+	+	+	+	+
	1% Sodium Lactate	+	+	+	+	+	+
	Fusidic acid	(−)	(−)	(−)	(−)	(−)	(−)
	D-Serine	+	+	+	b	+	+
	Troleandomycin	(−)	(−)	(−)	(−)	(−)	(−)
	Rifamycin SV	(−)	(−)	(−)	(−)	(−)	(−)
	Minocycline	(−)	(−)	(−)	(−)	(−)	(−)
	Lincomycin	(−)	(−)	(−)	(−)	(−)	(−)
	Guanidine HCl	(−)	(−)	(−)	(−)	(−)	(−)
	Niaproof 4	(−)	(−)	(−)	(−)	(−)	(−)
	Vancomycin	(−)	(−)	(−)	(−)	(−)	(−)
	Tetrazolium Violet	(−)	(−)	(−)	(−)	(−)	(−)
	Tetrazolium Blue	(−)	(−)	(−)	(−)	(−)	(−)
	Nalidixic Acid	+	+	+	+	+	+
	Lithium Chloride	+	+	+	+	+	+
	Potassium Tellurite	+	+	+	+	+	+
	Aztreonam	+	+	+	+	+	+
	Sodium Butyrate	+	+	+	+	+	+
	Sodium Bromate	b	b	(−)	b	b	b

+ = Positive; (−) = Negative; b = Borderline

The Biolog GEN III database identified isolates AN3, AN19, AN22 and BN7 as *S. haemolyticus*, with similarity indices (SIM) of 0.612 (0.529–0.720), 0.622 (0.524–0.674), 0.589 (0.582–0.596) and 0.66 (0.635–0.689). No identification was assigned to isolate AN25 after 48 h incubation. The closest match to AN25 was *S. haemolyticus* but the SIM value, 0.221 (0.171–0.267) was lower than the default cut-off of 0.5; precluding the assignment of an identification. Isolate FN2 was identified as *S. haemolyticus* (SIM = 0.525) on the first test whilst the subsequent two tests identified the isolate as *S. aureus* subsp. *aureus* (SIM = 0.646 and 0.682).

### Sequence analysis of housekeeping genes

Amplification and sequencing of the 16S rDNA gene was carried out in duplicate for each of the test isolates. Isolates AN19, AN22 and AN25 showed 99.8 to 100% homology with sequences deposited for *S. devriesei* in the European Bioinformatics Institute database (http://www.ebi.ac.uk). Both attempts at sequencing a partial fragment of the 16S rRNA gene of AN3, BN7, FN2 resulted in low quality sequences. No further attempts were made to analyze this data.

The *rpo*B gene sequences obtained for the six isolates under evaluation showed 100% homology with *S. devriesei* sequences in the database. Similarly, partial *hsp*60 sequences for five of the isolates (AN3, AN22, AN19, AN25, BN7) showed 100% homology with sequences deposited for *S. devriesei* whilst the *hsp*60 sequence for isolate FN2 showed 99.4% homology with *S. devriesei* sequences. Partial *dna*J sequences for the test isolates showed between 99.4 and 100% similarity with *S. devriesei* sequences. The species-level cut-off values previously reported for the 16S rRNA, *dna*J, *hsp*60, *rpo*B and *tuf* genes are 98.7, 97, 97, 94 and 97% respectively [8].

### Matrix-assisted laser desorption/ionization time of flight mass spectrometry analysis

**Fig. 1:**
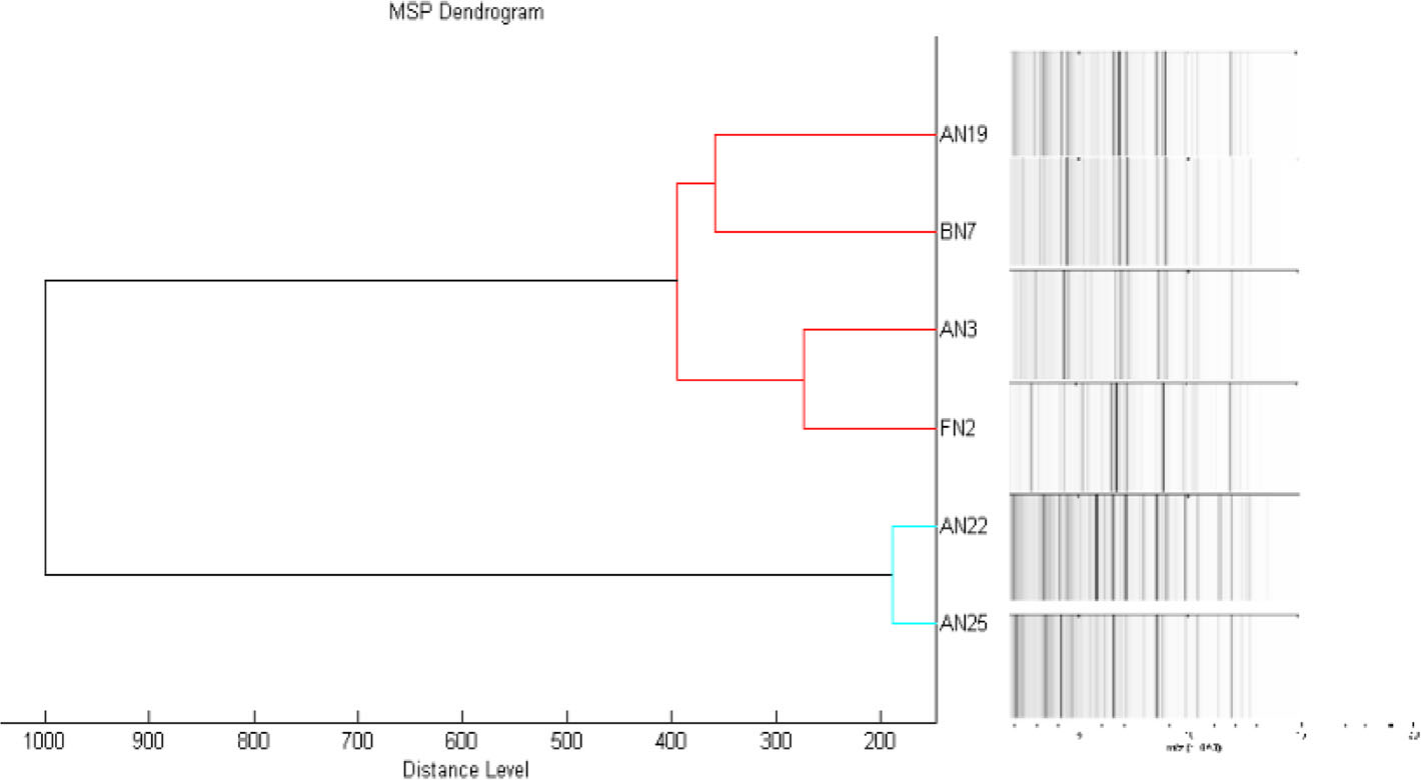
Minimal spanning tree generated by MALDI biotyper 3.0 software showing the six *Staphylococcus devriesei* isolates under investigation. The tree was created by the standard MALDI biotyper MSP creation method where distance values are relative and are always normalized to a maximum value of 1.00

The minimal spanning tree generated from the protein spectral fingerprints of the six CoNS isolates (Fig. 1) showed intra-species variation with isolates AN22 and AN25 clustering apart from the other four isolates. When the spectra were visualized in conjunction with the spectra of all *Staphylococcus* species contained in the MALDI biotyper database (not shown) the test isolates grouped together in a cluster comprising of *S. aureus*, *S. haemolyticus*, *S. hominis*, *S. hyicus* and *S. simulans*.

## Discussion

The sequencing of multiple housekeeping genes unequivocally identified the six isolates as *S. devriesei*. This *Staphylococcus* species was described 8 years ago by Belgian researchers following the characterization of several isolates recovered from bovine teat apices as well as a case of bovine subclinical mastitis [8]. Following the original description of the species only sporadic reports can be found in the literature relating the isolation of *S. devriesei*. Most of the reports document the isolation of this coagulase-negative *Staphylococcus* species from bovine teat skin, milk or milk by-products [19, 32–34]. A single report documenting the isolation of *S. devriesei* from a foot ulcer of a diabetic patient was found [35]. To the best of our knowledge this is the first report documenting the occurrence of *S. devriesei* in South Africa.

The six *S. devriesei* isolates were part of a collection of 102 CoNS isolates recovered from bovine IMIs in a previous study [31] *Staphylococcus chromogenes* was the predominant species identified in the study comprising almost 80% of the CoNS isolates. The *S. devriesei* isolates represent the second most common *Staphylococcus* species identified in the study with a prevalence of 5.9% (6/102). The *S. devriesei* isolates were recovered from lactating cows in three different herds. In order to assess the true prevalence and significance of this species as a causative agent of bovine IMIs, further investigations will need to be undertaken.

Preliminary phenotyping and molecular approaches used to identify the six CoNS, including a M-PCR assay, MALDI-TOF MS evaluation and *tuf* gene sequencing, failed to identify the isolates. Retrospectively, closer evaluation of the MALDI-TOF MS biotyper database (version 3.0) shows that there are no reference spectra for *S. devriesei*; accounting for the failure to achieve a successful identification. The current reference database comprises of only 38 *Staphylococcus* species with a number of the more recently described species not being represented including: *S. agnetis* (described in 2012), *S. argensis* (2015), *S. argenteus* (2015), *S. jettensis* (2013), *S. massiliensis* (2010), *S. microti* (2010), *S. petrasii* (2013), *S. rostri* (2010), *S. schweitzeri* (2015) and *S. stepanovicii* (2012) [9]. The accuracy of MALDI-TOF MS analysis, and essentially any commercially available phenotyping system, is wholly dependent upon the quality of the reference database used to match protein or phenotyping profiles. It is therefore imperative that regular updates are made to system databases to keep abreast with the description of new species as well as any changes in taxonomy.

Whilst MALDI-TOF MS was used in the initial study to identify the CoNS isolates, the technology was subsequently utilised to assess the relatedness of the six isolates as well as generate mass spectra which could be used to build up the local instrument database. Whilst all six isolates clustered together, sub-clusters were noted indicating intra-species variation with respect to cellular protein content. Unfortunately, with so few isolates available for evaluation it is difficult to make any inferences regarding the sub-clusters as no direct correlation can be seen between the clusters and the farm of origin or the biochemical profiles of the isolates.

To date, all reports documenting the isolation of *S. devriesei* have utilised genotyping methods to identify the bacterial isolates. The original description of the species by Supré and co-workers [8] provides the only information currently available concerning the phenotypic and biochemical diversity of this species. The six isolates characterized in this study conformed to the description provided by Supré and co-workers [8] in that, colonies of all isolates were surrounded by a zone of complete hemolysis. Furthermore, as noted by Supré and co-workers [8], variation with respect to the color of colonies was observed with some isolates presenting grey-yellow colonies whilst other isolates formed colonies which were yellow-orange in color.

The six *S. devriesei* isolates identified in this study were characterized using the Biolog GEN III MicroPlate™ (Biolog, USA) identification system to provide metabolic fingerprints for the field isolates and contribute to existing knowledge available concerning this species. In agreement with the *S. devriesei* type strain, KS-SP 60^T^ (=LMG 25332^T^ = CCUG 58238^T^), all six of the field isolates in this study had the ability to utilize D-fructose, D-galactose, α-D-glucose, glycerol, maltose, sucrose, D-trehalose and turanose. Five of the six isolates evaluated also utilized D-mannitol and α-D-lactose with the sixth isolate giving a borderline test reaction. The inability to utilize L-histidine, D-malic acid, p-hydroxy-phenylacetic acid, β-hydroxy-D,L-butyric acid, D-salicin and D-serine were consistent among the test isolates in this study. However, apart from D-salicin, no comparisons can be made with the report by Supré and co-workers [8], as the other substrates listed were not reported. Supré and co-workers [8] utilized the API50CH, API Staph ID 32 (bioMérieux, France) and the Staph Zym (Rosco, Denmark) phenotyping systems to characterize the original *S. devriesei* isolates. The profile of substrates incorporated into the aforementioned phenotyping systems are not the same as those included in the Biolog GEN III test system.

The Biolog GEN III database (version 2.3.1.404) currently comprises of data for only 35 of the 50 described *Staphylococcus* species. None of the *Staphylococcus* species described in the last decade, including *S. devriesei*, have been incorporated into the database so far. Consequently the assignment of a correct bacterial identification by the Biolog system was not expected for the six field isolates under evaluation. It is interesting to note however, that based upon the outcome of the carbon source utilization and chemical sensitivity assays, the Biolog database did, in fact, assign identifications to five of the six isolates. Most notably, four of the isolates were identified as *S. haemolyticus*. Phylogenetic studies [8, 10] have shown that *S. devriesei* is most closely related to *S. haemolyticus* and *S. hominis*. Consequently, the mis-identification of the isolates as *S. haemolyticus* is understandable. The similarity index values, which represent the level of agreement with the assigned identification, were low (0.589 to 0.66) indicating multiple mismatches with the profiles of the type strains in the database. However, since the SIM values were above the threshold of 0.5 used by the system, a positive identification was made. As was discussed for the MALDI-TOF MS system, the accuracy of any commercial bacterial identification system is underpinned by the quality of the database supporting the system. Failure to maintain current records, will negatively impact the accuracy and reliability of the system output. Diagnosticians and researchers should be cognisant of these shortcomings to avoid misidentification of bacterial isolates.

## Conclusions

To the best of our knowledge this is the first report documenting the isolation and analysis of *S. devriesei* from bovine IMIs in South Africa. Further sampling is required to ascertain the true prevalence and relative importance of *S. devriesei* as a mastitis causing pathogen in local dairy herds. The characterization studies carried out in this investigation using both MALDI-TOF MS and the Biolog GEN III Microplate™ system, will contribute to the limited knowledge currently available concerning this bacterial species.

## Methods

### Bacterial isolates

Six CoNS isolates, cultured from sub-clinical cases of bovine mastitis from three commercial dairy farms in KZN, were isolated in a previous study [31]. Preliminary bacteriological testing of the isolates, including the examination of isolates for coagulase production, the presence of clumping factor, as well as antimicrobial susceptibility testing of the isolates is described elsewhere [31].

### Phenotypic characterization of isolates

Isolates were streaked onto Columbia blood agar (Oxoid, England) supplemented with 5% sheep blood and the plates were incubated (Nuaire, USA) aerobically at 35 ± 1 °C. Bacterial colony morphology was documented after 24 and 48 h incubation. All six isolates were tested in triplicate using the Biolog GEN III MicroPlate™ identification system (Biolog, USA). Suspensions of the test isolates were prepared in inoculating fluid A (Biolog, USA) and the turbidity adjusted to between 90 and 98% transmittance using a turbidometer (Biolog, USA). One hundred microliters of the cell suspension was inoculated into each well of a GEN III microplate. This was repeated for each test suspension. The plates were incubated (Labotec, SA) at 33 °C. Microplates were read using the Biolog MicroStation™ after 24 and 48 h, and results were captured and analyzed by the system software (GEN III database, version 2.3.1.404).

### PCR amplification and sequencing of housekeeping genes

Genomic DNA was prepared from overnight cultures using the QIAGEN® DNeasy blood and tissue kit (Germany) according to the manufacturer’s instructions. Partial fragments of the 16S rRNA gene and three other housekeeping genes, *dna*J, *hsp*60 and *rpo*B were amplified as previously described [8, 16, 21, 36]. Details of the primers and PCR amplification conditions are summarized in Table 3. Separate PCR reactions mixtures were prepared for the amplification of each gene target. Each reaction mixture comprised of: 1X PCR buffer (Promega, USA); 4 mM MgCl_2_, (Promega, USA), 0.2 mM dNTPs (Promega, USA), 0.25 mM of each primer (Inqaba Biotechnical Industries, South Africa) and 1 U HotStart DNA polymerase (Promega, USA). Five microliters DNA template was added to each reaction mixture before amplification was carried out in a MJ Mini thermocycler (Bio-Rad, USA). Thermocycling conditions for all PCR assays are shown in Table 3.

**Table 3:** Amplification of the 16S rRNA, *dna*J, *hsp*60 and *rpo*B genes of the coagulase-negative *Staphylococcus* isolates

Gene target	Primer name	Oligonucleotide sequence^a^ (5′ – 3′)	PCR amplification conditions	Size of amplicon (bp)	Reference
16S rRNA	16F27 16R1522	AGAGTTTGATCCTGGCTCAG AAGGAGGTGATCCAGCCGCA	95 °C for 5 min, 30 cycles of [95 °C for 30 s, 58 °C for 60 s, 72 °C for 90 s] and a final extension step at 72 °C for 10 min	1500	[8]
*dna*J	SA-(F) SA-(R)	GCCAAAAGAGACTATTATGA ATTGYTTACCYGTTTGTGTACC	94 °C for 3 min, 5 cycles of [94 °C for 30 s, 45 °C for 30 s, 72 °C for 60 s], 30 cycles of [94 °C for 30 s, 50 °C for 30 s, 72 °C for 60 s], 72 °C for 3 min	920	[21]
*hsp*60	hsp60-F hsp60-R	CAIIIGCIGGIGAYGGIACIACIAC YKIYKITCICCRAAICCIGGIGCYTT	94 °C for 2 min, [94 °C for 60 s, TD^b^ for 2 min, 72 °C for 5 min], 20 cycles of [94 °C for 60 s, 35 °C for 2 min, 72 °C for 5 min], 72 °C for 10 min	600	[36]
*rpo*B	1418f 3554r	CAATTCATGGACCAAGC CCGTCCCAAGTCATGAAAC	94 °C for 5 min; 30 cycles of [94 °C for 45 s, 52 °C for 60 s, 72 °C for 90 s]; 72 °C for 10 min	899	[16]

^a^All primers were synthesized by Inqaba Biotechnical Industries (South Africa)

^b^TD = Touchdown PCR from 45 °C to 35 °C with temperature decrease of 0.5 °C per cycle; *I* Inosine, *K* Keto (G or T), *Y* pyrimidine (C or T)

The resulting PCR amplicons were electrophoresed in a 1.5% agarose gel (SeaKem® LE, Lonza, USA) with 1X Tris-borate-EDTA buffer (Melford, UK) at 100 V for 60 min. A 100 bp molecular weight marker (Thermo Scientific, USA) was included as a reference standard on all gels. Following staining in a 0.5 μg/mL ethidium bromide solution (10 mg/mL) (Bio-Rad, USA) gels were visualized under ultraviolet light (UVIPro, Uvitec, UK) and documented. The PCR amplicons were submitted to Inqaba Biotechnical Industries (South Africa) for sequencing. Trace data files were uploaded into CLC Genomics Workbench, version 6.0 (CLC, USA) and consensus sequences were generated. Consensus sequences were analyzed using the European Bioinformatics Institute website (http://www.ebi.ac.uk).

### Matrix-assisted laser desorption/ionization time of flight mass spectrometry

Overnight bacterial cultures (< 24 h) were submitted to the Department of Microbiology and Plant Pathology (University of Pretoria) for MALDI-TOF MS analysis (Bruker Daltonik GmbH, Germany). An ethanol/formic acid extraction was used to prepare isolates before ten spots were prepared on a steel target plate (MSP 96 polished-steel target) (Bruker Daltonik GmbH, Germany). Following air-drying, the spots were overlaid with 1 μL α-cyano-4-hydroxy-cinnamic acid (HCCA) solution (Bruker HCCA matrix portioned, Art. #255344). Three spectral readings were captured from each spot before being evaluated manually and analyzed using the MALDI biotyper software (version 3.0). Two minimal spanning trees were generated to (i) depict the relationship between the six CoNS under investigation (Fig. 1) and (ii) evaluate the relatedness of the test isolates to other *Staphylococcus* isolates in the MALDI biotyper reference library version 3.0 (Bruker Daltonik GmbH, Germany) based on spectral patterns (not shown).

## Abbreviations

CoNS: Coagulase-negative staphylococci
IMI: Intramammary infection
MALDI-TOF MS: Matrix-assisted laser/desorption ionization time of flight mass spectrometry
M-PCR: Multiplex polymerase chain reaction
SIM: Similarity indices
